# Nutritional Composition, Fatty Acid Content, and Mineral Content of Nine Sorghum (*Sorghum bicolor*) Inbred Varieties

**DOI:** 10.3390/foods13223634

**Published:** 2024-11-14

**Authors:** Paola Pontieri, Jacopo Troisi, Matteo Calcagnile, Fadi Aramouni, Michael Tilley, Dmitriy Smolensky, Marco Guida, Fabio Del Giudice, Antonio Merciai, Iryna Samoylenko, Alberto L. Chessa, Mariarosaria Aletta, Pietro Alifano, Luigi Del Giudice

**Affiliations:** 1 Sezione di Igiene, Istituto di Bioscienze e BioRisorse-UOS Napoli-CNR c/o Dipartimento di Biologia, 80134 Naples, Italy; felicita24@libero.it (F.D.G.); antonio.merciai7@gmail.com (A.M.); irynak@hotmail.it (I.S.); luigi.delgiudice@ibbr.cnr.it (L.D.G.); 2 Department of Medicine and Surgery, Theoreosrl, Spin off of the University of Salerno, Via Degli Ulivi, 3, 84090 Montecorvino Pugliano, Italy; troisi@theoreosrl.com; 3 Dipartimento di Medicina Sperimentale, Università del Salento, 73100 Lecce, Italy; matteo.calcagnile@unisalento.it (M.C.); pietro.alifano@unisalento.it (P.A.); 4 Center for Grain and Animal Health Research (CGAHR), Agricultural Research Service (ARS), United States Department of Agriculture (USDA), Manhattan, KS 66502, USA; fadi.aramouni@usda.gov (F.A.); michael.tilley@usda.gov (M.T.); dmitriy.smolensky@usda.gov (D.S.); 5 Department of Biology, University of Naples Federico II, 80126 Naples, Italy; marco.guida@unina.it; 6 Sorghum Breeder, Fernando Camino N_ 15 P4 F, 29016 Málaga, Spain; alberto.chessa@outlook.com; 7 DCSRSI SPR BIBLIOTECA, 80131 Naples, Italy; mariarosaria.aletta@igb.cnr.it

**Keywords:** sorghum pure lines, nutritional value, fatty acids, mineral elements

## Abstract

*Sorghum* is a self-pollinating species belonging to the Poaceae family characterized by a resistance to drought higher than that of corn. Sorghum (*Sorghum bicolor* L. Moench) has been grown for centuries as a food crop in tropical areas where it has an increasing importance, particularly as a cereal option for people with celiac disease. Over the past fifty years, food-grade varieties and hybrid seeds with white pericarp have been developed, particularly in the United States, to maximize sorghum food quality. Nutrient composition, including moisture, protein, carbohydrates, dietary fiber, fat content, fatty acid composition, and mineral content, was determined for nine inbred varieties with a stabilized food-grade sorghum genotype selected in the USA and grown under typical Mediterranean conditions. Differences in these nutritional components were observed among the varieties considered. Notable differences were found for monounsaturated and polyunsaturated fats, while saturated fatty acids were similar in all varieties. Oleic, linoleic, and palmitic acids were the most abundant fatty acids in all nine lines. Differences were also noted in mineral content, particularly for K, Mg, Al, Mn, Fe, Cu, Zn, and Ba. Enzyme-linked immunosorbent assays (ELISAs) demonstrated the absence of gliadin-like peptides in all the sorghum varieties analyzed, confirming, thus, that these analyzed varieties are safe for consumption by celiac patients. Knowledge of the nutritional values of sorghum lines is relevant for breeding programs devoted to sorghum nutritional content and for beneficial properties to human health.

## 1. Introduction

Sorghum (*Sorghum bicolor* L. Moench) is a self-pollinating crop mainly used as a staple food in Africa and Asia; sorghum seeds are often used as raw materials for alcoholic beverages, sweets, and as a source of glucose [1,2,3]. Sorghum is the fifth leading crop in the world after wheat, maize, rice, and barley [4]. The United States is the world’s largest producer and exporter of sorghum, generating roughly 20% of world sorghum production and nearly 80% of sorghum exports [2,3,5]. In several developing countries, sorghum has traditionally been used in food products and to prepare various health foods [3,6]. The crop is considered a source of safe food for celiac patients showing an immune reaction to gluten proteins found in several *Triticum* and closely related cereals [7]. Molecular evidence has directly demonstrated the absence of toxic gliadin-like peptides in sorghum, which can be considered safe for people with celiac disease [8]. This is why the potential role of sorghum in human health and disease prevention has gained increased attention in the past decade [1,2,3,9,10].

Recently, there has been a growing interest in sorghum as a food ingredient in Western societies: the use of sorghum in human food products has increased and expanded since early efforts in the United States to develop hybrids with white seeds (often called “food-grade” sorghum) for the production of gluten-free food [11]. Moreover, new technologies have been developed, aimed at enhancing the nutritional and functional values of sorghum proteins in industrial-scale processes [12]. Breeding programs of public and private institutions have released improved varieties adapted to semi-arid and tropical environments, including those that meet specific food and industrial requirements [11,13]. To meet the market demand, numerous collections of sorghum seeds have arisen all over the world, particularly in Ethiopia, China, and the United States, as well as at the International Crops Research Institute for the Semi-Arid Tropics (ICRISAT) [14]. With increasing world population and decreasing water supplies due to climate change, sorghum represents an important crop for future human use.

Thus, genetic improvement of sorghum through the crossing of pure lines is an important research area for increasing yield, disease resistance, environmental stress tolerance, and other desirable traits of sorghum. Moreover, the availability of pure lines with genetic diversity is essential for the effectiveness of crossing. The greater the genetic diversity among pure sorghum lines, the greater the chances of obtaining hybrids with improved characteristics. Generally, sorghum breeding programs aim to acquire know-how and technologies that allow for the cultivation, storage, and milling of new white sorghum hybrids for human consumption in an economical manner, making them appealing to farmers and end-users for gluten-free diets for celiac patients and healthy diets for everyone based on sorghum flour [3].

This study aimed to characterize nine inbred sorghum lines developed in the United States and grown in Mediterranean environments from a nutritional standpoint. The specific goals were to evaluate levels of lipids, fiber, total protein, carbohydrates, and minerals. To that end, the study of the nutritional composition of sorghum inbreds would help breeders select hybrids with superior quality and nutritional values. Furthermore, one of the aims of the study was to identify varieties with superior nutritional attributes and to demonstrate that all varieties were safe to produce foods suitable for consumption by celiac patients.

The aim of this study is to evaluate the nutritional properties and functional food traits of nine inbred sorghum varieties with a focus on their potential role in promoting health. We hypothesize that these varieties, especially those grown in the Mediterranean area, can serve as valuable sources of essential nutrients, such as proteins, carbohydrates, unsaturated fatty acids, and minerals. These varieties are anticipated to meet rising consumer demand for nutritious, gluten-free cereals and may support breeding programs focused on the development of high-quality, food-grade sorghum lines.

## 2. Materials and Methods

### 2.1. Sorghum Varieties

The sorghum inbreds and their respective seed sources used in this study are listed in Table 1. In 2021, an open field cultivation of sorghum was carried out in San Bartolomeo in Galdo (BN) located in the Fortore area of the Campania region, southern Italy (41°25′ N, 15°01′ E and 597 m.a.s.l.). The nine sorghum varieties were planted on April 2021, and the grain harvest took place on 28 October 2021. Monthly rainfall and maximum and minimum temperatures of the 2021 growing season recorded in the aforementioned area are demonstrated in Table 2, while soil physical–chemical properties of the aforementioned experimental area are shown in Table 3. The milling was carried out starting 1 month after the harvest of the sorghum grains which were stored in a dry environment at 16 °C.

### 2.2. Flour Sample Preparation

Roughly a kilogram of grain was pulverized into flour utilizing a two-roller mill from Chopin Moulin CD1 (Chopin S.A., Villeneuve la Garenne, France). Post-grinding, the samples underwent screening via a planetary sieve with a screen size measuring 120 μm^2^, manufactured by Buhler AG (Uzwil, Switzerland).

### 2.3. Moisture Content

The moisture content of the flour samples was measured as previously described [15]. Initially, a ceramic capsule was meticulously weighed following thorough desiccation at 100 °C under vacuum (25 mm Hg) using an oven (ISCO mod. NSV9035, Milan, Italy) and subsequently cooled down to room temperature inside a silica gel dryer. Then, a precisely weighed portion of the flour samples (approximately 2 g) was introduced into the dried ceramic capsule. The sample was then subjected to the same temperature and pressure conditions for around 5 h until a constant weight was achieved. At this point, the humidity had been extracted from the sample. The moisture content was calculated by determining the weight loss.

The moisture content of sorghum samples was calculated using the following equation:

$$Moisture\;\left(\%\right)=\frac{Weight\;of\;fresh\;sample-Weight\;after\;drying}{Weight\;of\;fresh\;sample}\times 100$$

### 2.4. Ash

Approximately 3 g of sorghum samples were weighed and placed in an incineration dish to determine the ash content. The dish was then ignited at roughly 550 °C and subsequently allowed to cool down within a desiccator. Once the dish had reached room temperature, it was immediately weighed, in line with the methodology established by AOAC [16] (AOAC, 1923).

For ash content, the following equation was used:

$$Ash\;\left(\%\right)=\frac{Weight\;of\;ash}{Initial\;sample\;weight}\times 100$$

### 2.5. Protein Content

Sorghum flour samples (2 g each) were analyzed for their nitrogen content by the AOAC [17] (AOAC, 1920) Kjeldahl method using a Mineral Six Digester and an Auto Disteam semi-automatic distillation unit (International PBI, Milan, Italy). The total protein content was subsequently calculated using a conversion factor of 6.25.

### 2.6. Total Lipid Content

The total lipid content was determined as previously described [18]. Initially, roughly 3 g of grain was ground down to a fine powder with liquid nitrogen, utilizing a mortar and pestle, and subsequently lyophilized using the FTS-System Flex-DryTM instrument. The ground whole meal was extracted for 4 h in a Soxhlet apparatus with chloroform (CHCl_3_). The resulting extracts were dried out with a rotary evaporator to obtain crude extracts, and the weight of the fat extracted was subsequently determined.

### 2.7. Gas Chromatography of Fatty Acids

To conduct the esterification of the fatty acids found within the crude extracts, and subsequent gas chromatographic analysis of the fatty acid methyl esters, the protocols established by Pontieri et al. [15,18] were followed. Initially, the solid sorghum fat was melted in an oven set to 50 °C. A single drop of fat was then transferred into a 1.5 mL vial, followed by the addition of 1 mL of hexane and 100 µL of 2 N KOH methanolic solution. The vial was then vortexed for a duration of 5 min before being left to stand undisturbed for an additional 5 min to allow for complete stratification of the hexanic portion, which contained the methyl ester of the fatty acids. Chromatographic separation was achieved via a GC-2010 (Shimadzu, Kyoto, Japan) that was fitted with a DB-Wax column (Phenomenex, Torrance, CA, USA), measuring 30 m in length, 0.25 mm in internal diameter, and with a 0.25 µm film thickness. The GC conditions utilized were as follows: carrier gas, helium; pressure, 75 kPa; injector temperature, 220 °C; FID temperature, 250 °C; and oven program, 170 °C for 8 min, 2 °C/min to 185 °C for 10 min, 1 °C/min to 190 °C for 12 min, and 10 °C/min to 240 °C for 5 min.

### 2.8. Carbohydrates

The quantity of carbohydrates present in the samples was calculated by deducting the values obtained for moisture, ash, protein, and fat content, as explained in the procedure described by Arienzo et al. [19].

### 2.9. Fiber Content

The AOAC method from 1995 [20] (AOAC, 1995) was employed to determine the fiber content. In this method, the sample was digested under acidic conditions using 0.255 N H_2_SO_4_, followed by alkaline digestion with 0.223 N NaOH in an automatic digestor (Velp Scientific mod. FIWE3, Usmate Velate, Monza e Brianza, Italy). The lost mass of the sample after incineration was considered as fiber.

### 2.10. Total Minerals Determination

The procedure described by Tenore et al. [21] and Pontieri et al. [22,23] was followed for the determination of the mineral elements of interest employing quadrupole inductively coupled plasma mass spectrometry (ICP-QMS) on an 820-MS instrument (Bruker Daltonics, Billerica, MA, USA). Operational parameters included the following: plasma flow rate: 18 L per minute; auxiliary flow rate: 1.8 L per minute; sheath gas flow rate: 0.14 L per minute; nebulizer flow rate: 0.98 L per minute; RF power: 1.40 kilowatts; pump rate: 4 revolutions per minute; stabilization delay: 20 s; and voltage settings for various extraction lenses and other components. Control of reactive contaminants was achieved through the use of high purity helium and hydrogen gases. The stability of plasma was maintained by employing high radio frequency power. All chemicals utilized were of the highest commercially available purity. Prior to utilization, all glassware and plastic containers underwent thorough cleaning with 10% ultra-pure grade nitric acid followed by rinsing with ultra-pure water. Calibration solutions were prepared from multi-elemental standard stock solutions with a concentration of 20.00 milligrams per liter. The calibration curves were generated using nine calibration solutions. Reagent blanks, consisting of ultra-pure water, were also analyzed to ensure the purity of reagents and laboratory equipment. The determination process involved the use of an internal standard mixture containing specific isotopes aspirated online alongside the sample and standard solution. Analysis included quantification of 17 isotopes using a calibration curve method, while others were quantified using a semi-quantitative approach based on mean response factors from adjacent elements.

For samples analysis, ash content was dissolved in a 5% HNO_3_ solution containing ultra-pure water, and the solution was filtered using regenerated cellulose filters that were free from ash.

### 2.11. ELISA Assay

The RIDASCREEN^®^ Gliadin standard test kit (Art. No R7001, R-Biopharm AG, Darmstadt, Germany) ELISA-based sandwich method was used to identify gliadins in grain flour samples according to both Valdés et al. [24] and the manufacturer’s instructions. Commercial gliadin standard 16–18% N (Sigma Aldrich, Milan, Italy) was used as the control.

### 2.12. Statistical Analysis

With the exception of total lipids analyses, which were performed in triplicate, all analyses were performed in quintuples (n = 5) (technical replicates), and the results are presented as mean ± SD. Data distributions were evaluated by the Shapiro–Wilk test. As all data were not normally distributed, differences in means were investigated using the Mann–Whitney non-parametric U test. This test is utilized to ascertain whether significant differences exist between two independent groups. Analysis of variance (ANOVA) was used to evaluate whether the different values were statistically significant or not. This test is employed to assess whether differences between group means stem from genuine distinctions in the groups themselves or if they are merely attributable to random variability. Tukey’s post-hoc test was used to identify which samples were different. This test was employed subsequent to conducting an ANOVA to discern which pairs of groups exhibit significant differences from each other. While ANOVA solely indicates the presence of overall differences between groups, Tukey’s post-hoc test aids in identifying the specific pairs of groups that differ significantly. A false discovery rate (FDR)-adjusted *p*-value was used to handle multiple comparisons. PAST 4 was employed to generate non-metrical multidimensional (NM-MDS) scaling based on the Bray–Curtis index [25].

## 3. Results

### 3.1. Weather Conditions and Soil Characteristics

The amount of rainfall during the crop growing season varied from 78 to 1000 mm (April–October) with a mean of 172.3 mm, mainly concentrated during spring and early autumn. Climatic results were measured in the year 2021 using a meteorological station located near the experimental field. Maximum and minimum temperatures, as well as precipitation, were collected monthly (Table 2) [26]. The soil of the area is mainly clayey, deep, and with a good water retention capacity, as reported in Table 3. It is well known that grain composition can vary significantly, influenced by the genotype and the growth environment, such as temperature, soil characteristics, fertilizers, and other factors. An example is the work published by Rooney (2004) [27], where it was demonstrated that high-nitrogen fertilizer levels increase grain protein content and decrease the amount of total carbohydrates.

### 3.2. Chemical Composition

The chemical composition of nine food-grade sorghum inbreds developed in the United States and grown in southern Italy is shown in Table 4. Protein content was higher in PL-2 and N3 inbreds and relatively lower in N2. The fat content was slightly higher in PL-1 than in PL-2, N3, N4, and N5, while a lower fat content was observed in PL-3, PL-4, Tw, and N2. Variations were also observed in the total carbohydrate content where Tw, N2, N3, and N5 showed a higher content than the other inbreds. Finally, a higher fiber content was evident for N4, PL-2, and PL-3 inbreds compared to the others. Particularly significant was the variation interval shown by the fiber content.

### 3.3. Fatty Acid Composition of Total Lipids

**Figure 1:**
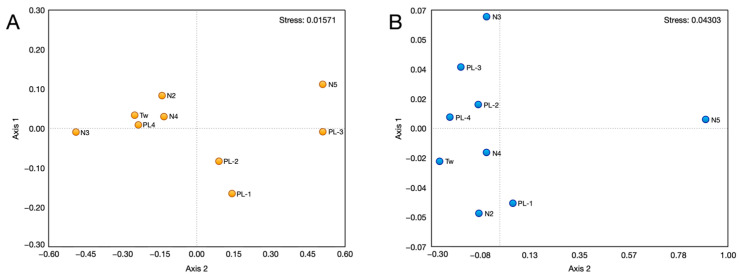
Non-metrical multidimensional scaling (NM-MDS) based on fatty acids content (**A**) and macronutrients, micronutrients, and trace elements (**B**).

The percentages of total fatty acids, as well as aggregated as saturated, monounsaturated, and polyunsaturated fats of the nine inbreds, are shown in Table 5. A higher content of total monounsaturated and polyunsaturated fats was found in both PL-1 and N5, while N3 showed a polyunsaturated fat content comparable to that found in PL-1. The saturated fatty acid content was almost similar in all varieties except for the N5 inbred which showed a higher content than the others. Oleic, linoleic, and palmitic acids were the most abundant fatty acids in all nine samples. Erucic acid was absent in six out of the nine lines. According to the NM-MDS (Figure 1A), the varieties N2, N4, Tw, and PL4 exhibit similarity in terms of fatty acid content. Variety N3 is not far from this first cluster and is also considered similar. Varieties PL2 and PL3 share similarity, as do PL-3 and N5.

### 3.4. Mineral Content

The results of the macro-elements, micro-elements, and trace elements of the nine inbreds of sorghum are reported in Table 6, Table 7, and Table 8, respectively. The content of macro-elements followed the sequence K > Mg > Ca > Na in the nine samples analyzed. The content of micro-elements followed the sequence Fe > Zn > Al > Mn >Cr >Cu> Ba > Ni > Pb > Mo > Ag > V > Sn > Co > As > Se >Be > Tl, while the content of trace elements followed the sequence Cd > Hg > U > Sb. Variations in the content of the elements were found among the nine inbreds of sorghum analyzed. On average, K and Mg were, respectively, the most abundant macro-elements, while Fe, Zn, Al, and Mn were the most abundant micro-elements and Cd, Hg, and U the most abundant trace elements (with some exceptions). Furthermore, the potassium content of the nine inbred samples averaged about 60 times higher than that of sodium. Notably, the K:Na ratio of each of the PL-1, N2, N3, and N4 inbreds was higher, while the K:Na ratio of the Tw inbred was lower than that of the other sorghum inbreds analyzed. The K:Na ratio was higher than the recommended ratio 5.0 [26] for human diet. The high K:Na ratio suggests that sorghum inbreds may be suitable for improving health problems due to sodium retention. In fact, diets with a higher K:Na ratio are recommended for particular patients [28].

According to the NM-MDS (Figure 1B), the N5 variety exhibits distinct characteristics in terms of macronutrients, micronutrients, and trace elements compared to all other samples. Despite variations in some features, such as Ni and Co content, the remaining varieties share common characteristics.

Our results for Mg, K, and Zn were lower than those reported recently by Jacimovic et al. [29]. On the contrary, our Fe levels were much higher (Table 9). Mineral content of grains is affected by factors such as grain variety, soil composition, and weather conditions. This might explain the differences in the values between the two studies.

### 3.5. Immunochemical Evidence for the Absence of Gluten in Sorghum Inbreds

The results of immunochemical measurement of gliadin concentration in the sorghum flour from all samples tested demonstrated that gluten levels in all sorghum inbreds were less than 5 ppm (the detectable limit is 5 ppm) (Table 10). Those values are well below the 20 ppm threshold that has been proposed to be safe for celiac patients [24].

## 4. Discussion

Depending on the region of cultivation, the type of sorghum and the purpose of its production vary greatly, and the primary focus of sorghum breeders around the world is to improve nutritional and quality traits, yield, maturity, and adaptability [11,13,27]. In recent years there has been a growing interest worldwide in both functional and nutraceutical foods, including sorghum, and research has focused on identifying the mechanisms associated with their preventive or therapeutic potential [10]. As a consequence, the focus of sorghum breeding is the development of hybrid varieties characterized by high nutritional value and quality and by high yield, high adaptability, early maturation, and resistance tolerance to disease, pests, and stress [11,27,30].

With the aim of obtaining higher-quality sorghum cultivars, genetic improvement programs should be directed towards the use of pure lines of sorghum whose nutritional properties are known, following a genetic strategy to predict hybrids with desired nutritional and qualitative parameters [31,32]. Sorghum exhibits high genetic diversity, with many thousands of accessions and landraces collected and developed worldwide, particularly in collections in the USA, Ethiopia, China, and at the International Crops Research Institute for the Semi-Arid Tropics (ICRISAT) [14]. It has been demonstrated that grain composition can vary significantly due to genetic and environmental factors [15,18,22,27,33]. Inbred varieties and hybrids exhibit significant variations in yield potential, adaptability, and grain-quality characteristics; thus, cultivar selection is one of the most important considerations in crop management [11]. The genetic diversity of sorghum provides an opportunity to enhance the crop at the genetic level in interaction with the most suitable growth environment.

Currently, around the world, a wide range of genetic diversity of sorghum is available. However, inbred lines for the development of hybrids with improved nutritional and functional properties have not yet been fully evaluated [34]. Due to the growing interest in sorghum as a functional food and nutraceutical for human health [35], we have analyzed the nutritional composition, fatty acid content, and mineral content of nine food-grade sorghum lines grown under Mediterranean conditions, in an area with predominantly clayey soil, deep, and with good water retention capacity, that is, a suitable environment for the selection of inbred lines with high agronomic value and high nutritional quality.

Among the inbreds analyzed, those with the best characteristics could be used for hybrid development and germplasm improvement.

The composition profiles of the nine inbreds had some differences in both protein and carbohydrate percentages. As described in the Results section, PL-2 and N3, having a higher content of protein and fat, could be used in inbred breeding. Additionally, the higher fiber content of N4, PL-3, PL-4, PL-1, and particularly PL-2 may have health benefits beyond those conferred by the high protein content.

The amounts of total saturated fat were similar in the genotypes considered, but much lower than the values of total monounsaturated and polyunsaturated fats. Inbred varieties PL-1, PL-2, and N5 had higher amounts of both monounsaturated and polyunsaturated fatty acids, so these three lines may have a slight nutritional advantage. Linoleic, oleic, and palmitic acids were predominant over other fatty acids in all inbreds. Unsaturated fatty acids are important for human nutrition, as they are main components of biological membranes and play a role in modulating membrane fluidity. Furthermore, unsaturated fatty acids, unlike those saturated, do not have a cholesterogenic property and reduce the risk of thrombosis. Due to these characteristics, unsaturated fatty acids are strongly recommended to reduce the risk of atherosclerosis [10].

The content of each macro-element measured shows that the primary minerals are K, followed by Mg, a finding consistent with the available literature [36].

Furthermore, while the concentrations of the macro-elements were similar among all inbreds, both Ca and Na were higher in the Tw and lower in the PL-1 lines than in other inbreds. These data support previous studies indicating that the mineral content of sorghum is influenced by both genetic and environmental factors [33]. With regards to the macro-element content, we found a K:Na ratio higher than that recommended in the human diet for varieties of sorghum [26]. A higher K:Na ratio may improve bone health, reduce muscle loss, and moderate other chronic diseases such as hypertension [28].

Furthermore, it has been suggested that the K:Na ratio is an indicator of the quality of the diet of pregnant women and that the Na/K ratio in community settings is a potential population-based approach to address hypertension [37,38]. Prioritizing potassium-rich foods and limiting sodium intake can improve overall health outcomes, especially in reducing risks associated with chronic conditions.

It can also be noted from our data that the magnesium content was higher than typically found in corn (on average, 0.47 g kg^−1^) and wheat flour (on average, 0.25 g kg^−1^) [39]. Due to their high magnesium content, the inbreds considered in this study can be a good source of magnesium. Magnesium is an important macro-element necessary for the function of several enzyme systems [36].

In the nine inbreds, differences were noted in the concentration of micro-elements that could be due to the sorghum genotype, to soil conditions, and to the plant maturity state at harvest [23,40]. Fe, the most abundant micro-element, is an essential micro-element in human nutrition, and its deficiency is a serious public health threat worldwide. The expanding production of sorghum for human use in the United States and in the Mediterranean countries [3] is also motivated by the high levels of Fe in this crop, and by the beneficial nutritional effects of this micro-element. The results reported in the present study show a high content of Zn in all inbreds considered. This finding is notable because Zn deficiencies are a public health concern worldwide. It is important to underline that, as regards the trace elements, their concentration in all nine inbreds analyzed in this study did not exceed the maximum allowed by Regulation (CE) n. 41/2009. The nine inbred varieties of sorghum used in this study each have their own stabilized genetic heritage, which differs from one another, and they were all grown in the same typical Mediterranean environment. The integrity of the results, which report differences in nutritional composition, fatty acids, and mineral content, is ensured by previous studies that have demonstrated that the composition of sorghum grain can vary significantly due to genetic and environmental growth factors [15,18,22,27,33].

Finally, in this study, the nine inbreds we have characterized were also tested for gliadin concentration to confirm previous reports on the safety of sorghum for people with celiac disease [8].

The PL-2 and N3 genotypes have a high content of proteins, carbohydrates, fibers, unsaturated fatty acids, and minerals and appear to be the most suitable for the selection of hybrids with high nutritional quality. Substantial research has been conducted with the aim of developing the cultivation of sorghum lines in the Mediterranean area for use in the production of human food products [15,18,33].

The aim of this research was achieved, mainly comparing the nutritional composition of inbred sorghum varieties in order to assess the variation in nutritional properties and identify varieties with improved nutritional characteristics, thus providing greater potential health benefits for consumers. Furthermore, this research contributes to the body of knowledge on the nutritional composition of sorghum, particularly for sorghum varieties grown outside of the main sorghum breeding regions.

## 5. Conclusions

Today, particularly in developed countries, there is a significant demand for functional and nutraceutical healthy food. Adopting a diet based on food-grade white sorghum can promote disease prevention, leading to substantial cost savings for national health services. Food-grade sorghum is increasingly vital in the developed world, particularly as a cereal option for individuals with celiac disease. When developing food-grade sorghum varieties, several key grain traits, including grain quality, are considered. The functional food and feed traits and characteristics of sorghum (e.g., pasting, temperature, taste, amylase activity, shelf-life, etc.) are not fully understood. However, studies on cereal crops, including sorghum, suggest that these traits may be influenced by both genetic characteristics and the growth environment.

Breeding programs of both public and private institutions have released improved varieties adapted to semi-arid and tropical environments, including those that meet specific food and industrial requirements. To meet market demand, numerous collections of sorghum seeds have emerged worldwide, particularly in Ethiopia, China, and the United States, as well as at the International Crops Research Institute for the Semi-Arid Tropics (ICRISAT). The present study supports the strategy of evaluating sorghum nutritional properties such as protein and carbohydrate contents, levels of unsaturated fatty acids, and minerals of nine inbred sorghum varieties developed in the USA and grown in the Mediterranean area. The current research provides valuable information on the nutrient composition of various inbred sorghum varieties and supports the burgeoning sorghum breeding programs focused on the unique health benefits of consuming whole grain sorghum. The findings of this study underscore the importance of developing sorghum inbred lines with superior quality traits for use in breeding programs worldwide. These programs aim to select new sorghum hybrids that align with the preferences and nutritional needs of end-consumers, thereby ensuring a safe and healthy diet.

## Figures and Tables

**Table 1:** List of Sorghum inbreds grown in Italy.

Inbreds	Type
PL-1 = TX436	Normal food grade
PL-2 = 05MN5113	Normal food grade
PL-3 = 05MN5115	Normal food grade
PL-4 = Macia	Normal food grade
Tw = B.TXARG-1	Waxy food grade
N2 = SURENO	Normal food grade
N3 = DORADO	Normal food grade
N4 = R.TX436	Normal food grade
N5 = SEPON82	Normal food grade

The source of the seeds was Purdue University (M. Tunistra, Indiana, USA). These varieties are not currently available for commercial use.

**Table 2:** Monthly rainfall and maximum and minimum temperatures of the 2021 growing season recorded at San Bartolomeo in Galdo, Italy.

Month	T. Min (°C)	T. Max (°C)	T. Mean (°C)	Rainfall (mm)
April	7.8	14.4	11.1	78
May	9.8	18.6	14.2	30.8
June	14.1	27.8	20.9	29
July	18.8	28.5	23.6	15.2
August	17.4	29.4	23.4	29
September	14.7	23.4	19.0	24.6
October	10.8	13.4	12.1	1000
	Mean	Mean	Mean	Total
	13.3	22.2	17.7	172.3

**Table 3:** Physico-chemical properties of the soil of the experimental field located in San Bartolomeo in Galdo (BN), Campania (Italy).

Scheme 0	0–60 cm Depth
Clay (%)	42.6
Silt (%)	18.8
Sand (%)	39.4
pH	8.4
Exchangeable Ca (g/kg)	119
Available P (mg/kg)	16
Exchangeable K (meq/100 g)	1.4
Exchangeable Mg (meq/100 g)	1.6
Total Ca carbonate (g/kg)	68
Total N (g kg^−1^)	0.8
CSC (meq/100 g)	28
Organic C (g kg^−1^)	2.5

**Table 4:** Nutritional composition of 9 food-grade sorghum inbred varieties grown under typical Mediterranean conditions.

Parameter	Moisture (%)	Ash (%)	Total Proteins (%)	Fats (%)	Total Carbohydrates (%)	Sugars (%)	Fibers (%)
PL-1	12.2 ± 0.9 ^dg^	1.7 ± 0.1 ^bcdeg^	11 ± 0.4 ^fg^	2.93 ± 0.12 ^cdefh^	68.74 ± 2.75	1.6 ± 0.2 ^f^	3.43 ± 0.14 ^bcdefghi^
PL-2	11.7 ± 0.6 ^dg^	2 ± 0.2 ^acdf^	12.2 ± 1.2 ^cdfi^	2.61 ± 0.29	65.9 ± 5.27	1.7 ± 0.2 ^efg^	5.59 ± 0.56 ^acdefghi^
PL-3	12.3 ± 0.6 ^dgi^	2.5 ± 0.3 ^abfhi^	10.5 ± 0.4 ^bfg^	2.3 ± 0.07 ^afghi^	65.54 ± 5.24	1.5 ± 0 ^df^	6.86 ± 0.69 ^abdefgi^
PL-4	10.6 ± 0.7 ^abc^	2.4 ± 0.2 ^abfhi^	10.7 ± 0.5 ^bfg^	2.45 ± 0.27 ^a^	69.41 ± 6.25	1.8 ± 0.2 ^cefgi^	4.44 ± 0.13 ^abcefghi^
Tw	11.8 ± 0.8 ^g^	2.3 ± 0.2 ^afhi^	10.7 ± 0.6 ^fg^	2.36 ± 0.09 ^ah^	71.15 ± 6.4	1.4 ± 0.1 ^bdh^	1.69 ± 0.08 ^abcdfghi^
N2	11.2 ± 1.3	1.7 ± 0.1 ^bcdeg^	9 ± 0.3 ^abcdegh^	2.47 ± 0.1 ^ac^	72.87 ± 8.74	1.3 ± 0.1 ^abcdhi^	2.76 ± 0.3 ^abcdeghi^
N3	10.5 ± 0.4 ^abce^	2.2 ± 0.1 ^afhi^	12.5 ± 0.4 ^acdefhi^	2.63 ± 0.26 ^c^	70.74 ± 6.37	1.4 ± 0.1 ^bdh^	1.43 ± 0.17 ^abcdefhi^
N4	11.4 ± 1	1.9 ± 0.2 ^cdeg^	10.8 ± 0.6 ^fg^	2.63 ± 0.11 ^ace^	65.54 ± 1.97 ^i^	1.6 ± 0.1 ^efg^	7.73 ± 0.85 ^abdefgi^
N5	10.9 ± 0.9 ^c^	1.8 ± 0.1 ^cdeg^	10 ± 1.2 ^bg^	2.66 ± 0.27 ^c^	73.47 ± 6.61 ^h^	1.5 ± 0.1 ^df^	1.17 ± 0.09 ^abcdefgh^
Int.Var ^1^	10.5–12.3	1.7–2.5	9.03–12.5	2.3–2.93	65.54–73.47	1.3–1.8	1.17–7.73

^1^ Interval of variation. The letter a in superscript indicates a value significantly different compared to the PL-1 variety, b compared to PL-2 variety, c compared to PL-3 variety, d compared to PL-4 variety, e compared to Tw variety, f compared to N2 variety, g compared to N3 variety, h compared to N4 variety, i compared to N5 variety.

**Table 5:** Fatty acid content of 9 food-grade sorghum inbred varieties grown under typical Mediterranean conditions. The letter a in superscript indicates a value significantly different compared to the PL-1 variety, b compared to PL-2 variety, c compared to PL-3 variety, d compared to PL-4 variety, e compared to Tw variety, f compared to N2 variety, g compared to N3 variety, h compared to N4 variety, i compared to N5 variety.

Parameter	PL-1	PL-2	PL-3	PL-4	Tw	N2	N3	N4	N5	Int.Var ^4^
Myristic C14:0	0.082 ± 0.003 ^bcdefghi^	0.054 ± 0.004 ^ai^	0.05 ± 0.003 ^af^	0.0 5± 0.002 ^af^	0.05 ± 0.004 ^af^	0.057 ± 0.003 ^acdeghi^	0.05 ± 0.003 ^afhi^	0.049 ± 0.003 ^af^	0.046 ± 0.004 ^abf^	0.05–0.082
Palmitic C16:0	11.58 ± 0.58 ^defghi^	11.84 ± 0.59 ^defghi^	11.88 ± 0.59 ^defghi^	13.47 ± 0.53 ^abc^	13.46 ± 1.07 ^abc^	14.22 ± 0.56 ^abchi^	13.45 ± 0.94 ^abc^	13.40 ± 0.40 ^abcf^	13.26 ± 0.53^abcf^	11.58–13.47
Palmitoleic C16:1 9 c	0.291 ± 0.015 ^bdefghi^	0.215 ± 0.009 ^acdefghi^	0.31 ± 0.009 ^bdefghi^	0.34 ± 0.01 ^abcefghi^	0.600 ± 0.018 ^abcdghi^	0.567 ± 0.028 ^abcdghi^	0.369 ± 0.011 ^abcdefhi^	0.516 ± 0.021 ^abcdefg^	0.500 ± 0.025 ^abcdefg^	0.215–0.600
Margaric C17:0	0.082 ± 0.007 ^bcdegi^	0.046 ± 0.002 ^acefghi^	0.070 ± 0.004 ^abd^	0.050 ± 0.004 ^acefghi^	0.070 ± 0.004 ^abd^	0.073 ± 0.004 ^bd^	0.069 ± 0.004 ^abd^	0.074 ± 0.002 ^bd^	0.07 ± 0.004 ^abd^	0.046–0.082
Margaroleic C17:1 10 c	0.08 ± 0.00 ^bcdfgh^	0.06 ± 0.00 ^acdfh^	0.07 ± 0.00 ^abdfgh^	0.071 ± 0.00 ^abcfgh^	0.07 ± 0.01 ^h^	0.062 ± 0.00 ^abcdgh^	0.06 ± 0.00 ^acdfh^	0.00 ± 0.00 ^abcdefgi^	0.07 ± 0.01 ^h^	0.00–0.08
Stearic C18:0	1.22 ± 0.10 ^f^	1.33 ± 0.05 ^cdefhi^	1.22 ± 0.08 ^bf^	1.205 ± 0.07 ^bf^	1.21 ± 0.08 ^bf^	1.08 ± 0.03 ^abcdeghi^	1.24 ± 0.07 ^f^	1.24 ± 0.03 ^bf^	1.18 ± 0.07 ^bf^	1.08–1.33
Oleic C18:1 9c	35.92 ± 1.07 ^cdefghi^	35.08 ± 2.80 ^cdegi^	41.06 ± 2.46 ^abdefgh^	30.65 ± 2.45 ^abci^	30.39 ± 0.91 ^abcgi^	31.97 ± 2.55 ^acgi^	27.61 ± 2.20 ^abcefhi^	32.33 ± 1.94 ^acgi^	40.95 ± 2.04 ^abdefgh^	27.61–41.06
Linoleic C18:2 9c12c	46.95 ± 1.40 ^cdegi^	47.79 ± 1.91 ^cdegi^	42.54 ± 2.12 ^abdefgh^	50.76 ± 1.52 ^abci^	50.98 ± 1.52 ^abci^	49.38 ± 2.96 ^ci^	54.28 ± 4.34 ^abci^	49.59 ± 3.96 ^ci^	40.66 ± 1.22 ^abdefgh^	42.54–54.28
Linolenic C18:3 c9c12c15	2.55 ± 0.17 ^bcdefghi^	2.14 ± 0.08 ^acefgh^	1.49 ± 0.04 ^abdehi^	2.05 ± 0.10 ^acefgh^	1.83 ± 0.05 ^abcdfgi^	1.45 ± 0.10 ^abdehi^	1.57 ± 0.07 ^abdei^	1.71 ± 0.13 ^abcdfi^	2.01 ± 0.10 ^acefgh^	1.45–2.55
Arachidic C20:0	0.14 ± 0.01 ^bcegi^	0.16 ± 0.01 ^af^	0.17 ± 0.01 ^adfh^	0.15 ± 0.01 ^c^	0.16 ± 0.01 ^af^	0.14 ± 0.01 ^bcegi^	0.16 ± 0.01 ^af^	0.151 ± 0.01 ^c^	0.16 ± 0.01 ^af^	0.14–0.17
Eicosenoic C20:1 11c	0.220 ± 0.009 ^bdgi^	0.160 ± 0.008 ^acdefghi^	0.220 ± 0.009 ^bdgi^	0.200 ± 0.014 ^abcfghi^	0.210 ± 0.017 ^bfgi^	0.238 ± 0.017 ^bde^	0.250 ± 0.015 ^abcde^	0.229 ± 0.011 ^bdi^	0.250 ± 0.008 ^abcdeh^	0.160–0.250
Behenic C22:0	0.160 ± 0.005 ^bcdefghi^	0.077 ± 0.002 ^acdefghi^	0.130 ± 0.007 ^abdefghi^	0.110 ± 0.009 ^abcefghi^	0.040 ± 0.003 ^abcdfghi^	0.070 ± 0.005 ^abcdeghi^	0.070 ± 0.003 ^abcdehi^	0.060 ± 0.002 ^abcdefg^	0.060 ± 0.002 ^abcdefg^	0.040–0.160
Lignoceric C24:0	0.067 ± 0.005 ^cdefghi^	0.060 ± 0.004 ^cdefgh^	0.040 ± 0.001 ^abdefghi^	0.110 ± 0.004 ^abcei^	0.12 ± 0.006 ^abcdfi^	0.102 ± 0.008 ^abcegi^	0.120 ± 0.01 ^abcfi^	0.110 ± 0.009 ^abci^	0.060 ± 0.002 ^acdefgh^	0.040–0.120
Erucic C22:1 13c	0.000 ± 0.000 ^dgh^	0.000 ± 0.000 ^dgh^	0.000 ± 0.000 ^dgh^	0.011 ± 0.000 ^abcefghi^	0.000 ± 0.000 ^dgh^	0.000 ± 0.000 ^dgh^	0.008 ± 0.000 ^abcdefhi^	0.003 ± 0.000 ^abcdefgi^	00.000 ± 0.000 ^dgh^	0.00–0.008
MSF ^1^	1.07 ± 0.11 ^defghi^	0.93 ± 0.07 ^defgi^	0.96 ± 0.07 ^defgi^	0.77 ± 0.07 ^abci^	0.74 ± 0.04 ^abcfhi^	0.81 ± 0.03 ^abcegi^	0.74 ± 0.04 ^abcfhi^	0.87 ± 0.07 ^aegi^	1.53 ± 0.09 ^abcdefgh^	0.74–1.53
PSF ^2^	1.47 ± 0.15 ^cef^	1.33 ± 0.15 ^ci^	1.03 ± 0.07 ^abdefghi^	1.32 ± 0.13 ^cgi^	1.27 ± 0.05 ^acgi^	1.27 ± 0.06 ^acgi^	1.49 ± 0.06 ^cef^	1.37 ± 0.12 ^ci^	1.59 ± 0.14 ^bcdefh^	1.03–1.59
SF ^3^	0.39 ± 0.01 ^bcdei^	0.35 ± 0.02 ^acghi^	0.31 ± 0.02 ^abdfghi^	0.37 ± 0.01 ^aci^	0.35 ± 0.03 ^agi^	0.39 ± 0.04 ^ci^	0.4 ± 0.03 ^bcei^	0.39 ± 0.03 ^ci^	0.54 ± 0.04 ^abcdefgh^	0.31–0.54

^1^ Monounsaturated fats; ^2^ polyunsaturated fats; ^3^ saturated fats, ^4^ interval of variation.

**Table 6:** Nutritionally essential macro-element content of 9 food-grade sorghum inbred varieties grown under typical Mediterranean conditions (mg kg^−1^). The letter a in superscript indicates a value significantly different compared to the PL-1 variety, b compared to PL-2 variety, c compared to PL-3 variety, d compared to PL-4 variety, e compared to Tw variety, f compared to N2 variety, g compared to N3 variety, h compared to N4 variety, i compared to N5 variety.

Parameter	Na	Mg	K	Ca
PL-1	0.14 ± 0.01 ^bcdefghi^	6.16 ± 0.37 ^bcdg^	13.66 ± 0.82 ^g^	0.51 ± 0.03 ^bcdefghi^
PL-2	0.24 ± 0.01 ^acdefgh^	7.06 ± 0.42 ^aefghi^	14.04 ± 0.7 ^g^	0.82 ± 0.04 ^adefghi^
PL-3	0.32 ± 0.01 ^abdefghi^	7.32 ± 0.37 ^aefghi^	14.25 ± 1 ^g^	0.83 ± 0.04 ^adefghi^
PL-4	0.29 ± 0.01 ^abcefghi^	6.99 ± 0.21 ^aefghi^	14.09 ± 0.56 ^g^	0.96 ± 0.06 ^abcefhi^
Tw	0.35 ± 0.01 ^abcdfghi^	6.05 ± 0.24 ^bcdg^	13.04 ± 0.91 ^gh^	1.73 ± 0.12 ^abcdfghi^
N2	0.19 ± 0.01 ^abcdegi^	5.88 ± 0.29 ^bcdg^	14.38 ± 1.01 ^eg^	0.72 ± 0.02 ^abcdeghi^
N3	0.21 ± 0.01 ^abcdefi^	8.2 ± 0.25 ^abcdefhi^	16.38 ± 0.82 ^abcdefhi^	0.99 ± 0.03 ^abcefhi^
N4	0.2 ± 0.01 ^abcdei^	5.91 ± 0.35 ^bcdg^	14.54 ± 0.87 ^eg^	0.68 ± 0.02 ^abcdefgi^
N5	0.25 ± 0.01 ^acdefgh^	6.3 ± 0.44 ^bcdg^	14.14 ± 0.42 ^g^	0.63 ± 0.03 ^abcdefgh^
Int.Var ^1^	0.14–0.35	5.88–8.2	13.04–16.38	0.51–1.73

^1^ Interval of variation.

**Table 7:** Nutritionally essential micro-element content of 9 food-grade sorghum inbred varieties grown under typical Mediterranean conditions (mg kg^−1^).

Parameter	PL-1	PL-2	PL-3	PL-4	Tw	N2	N3	N4	N5	Int.Var ^1^
Be	<0.01	0.02 ±0.00	0.03 ±0.00	0.04 ±0.00	0.05 ±0.00	0.02 ±0.00	0.02 ±0.00	0.02 ±0.00	0.02 ±0.00	<0.01–0.05
Al	13.91 ± 0.83 ^bcdefghi^	65.47 ± 3.93 ^acdefghi^	107.15 ± 3.21 ^abdefghi^	131.88 ± 5.28 ^abcefghi^	178.44 ± 8.92 ^abcdfghi^	54.42 ± 3.27 ^abcdeghi^	42.62 ± 1.7 ^abcdefhi^	56.77 ± 3.97 ^abcdefgi^	25 ± 1.75 ^abcdefgh^	13.91–178.44
V	<0.01 ^bcdef^	<0.01 ^acdefghi^	0.15 ± 0.01 ^abdefghi^	0.22 ± 0.01 ^abcefghi^	0.35 ± 0.02 ^abcdfghi^	0.08 ± 0.00 ^abcdeghi^	0.04 ± 0.00 ^bcdef^	<0.01 ^bcdef^	<0.01 ^bcdef^	<0.01–0.35
Cr	5.47 ± 0.38 ^bcdefghi^	17.79 ± 0.53 ^acdefghi^	3.67 ± 0.18 ^abefghi^	3.55 ± 0.18 ^abefghi^	4.18 ± 0.21 ^abcdfghi^	4.69 ± 0.14 ^abcdeghi^	3.07 ± 0.09 ^abcdefhi^	6.91 ± 0.21 ^abcdefgi^	5.71 ± 0.34 ^bcdefgh^	3.07–17.79
Mn	36.33 ± 2.18 ^bcdeg^	41.78 ± 2.92 ^acdgh^	55.12 ± 3.31 ^abdefhi^	47.39 ± 3.32 ^abcfghi^	46.53 ± 3.26 ^acfghi^	40.09 ± 2.81 ^cdegh^	56.79 ± 1.7 ^abdefhi^	34.95 ± 1.75 ^bcdefg^	37.54 ± 2.63 ^cdeg^	34.95–56.79
Fe	113.17 ± 6.79 ^cdefgh^	117.2 ± 8.2 ^cdefh^	154.25 ± 4.63 ^abdefghi^	182.04 ± 5.46 ^abcfghi^	193.66 ± 13.56 ^abcfghi^	135.67 ± 5.43 ^abcdei^	128.12 ± 6.41 ^acdei^	132.68 ± 9.29 ^abcdei^	113.42 ± 6.81 ^cdefgh^	113.17–193.66
Co	0.06 ± 0.00 ^bcdefhi^	0.07 ± 0.00 ^adefghi^	0.07 ± 0.00 ^adefghi^	0.08 ± 0.00 ^abceghi^	0.11 ± 0.01 ^abcdfgh^	0.08 ± 0.00 ^abceghi^	0.06 ± 0.00 ^bcdefhi^	0.05 ± 0.00 ^abcdefgi^	0.12 ± 0.01 ^abcdfgh^	0.05–0.12
Ni	1.89 ± 0.06 ^bcdefghi^	1.45 ± 0.1 ^acdfi^	0.95 ± 0.03 ^abdefghi^	1.32 ± 0.04 ^abcefi^	1.61 ± 0.1 ^acdfghi^	2.37 ± 0.09 ^abcdeghi^	1.40 ± 0.07 ^acefi^	1.40 ± 0.06 ^acefi^	3.9 ± 0.23 ^abcdefgh^	0.95–2.37
Cu	11.05 ± 0.66 ^bcdefghi^	16.15 ± 0.97 ^adefghi^	16.39 ± 0.98 ^adefghi^	13.77 ± 0.41 ^abcfghi^	13.67 ± 0.68 ^abcfghi^	9.26 ± 0.46 ^abcdegi^	24.13 ± 0.72 ^abcdefhi^	9.46 ± 0.38 ^abcdegi^	33.79 ± 2.37 ^abcdefgh^	9.26–33.79
Zn	97.85 ± 3.91 ^bcdghi^	107.93 ± 5.4 ^aefgi^	107.76 ± 6.47 ^aefgi^	107.68 ± 3.23 ^aefgi^	96.59 ± 3.86 ^bcdghi^	93.9 ± 6.57 ^bcdghi^	130.44 ± 6.52 ^abcdefhi^	113.06 ± 4.52 ^aefgi^	578.52 ± 28.93 ^abcdefgh^	93.9–578.52
As	0.03 ± 0.00 ^bcdefghi^	0.09 ± 0.00 ^adefghi^	0.09 ± 0.00 ^adefghi^	0.10 ± 0.00 ^abcfgi^	0.10 ± 0.00 ^abcfgi^	0.05 ± 0.00 ^abcdeh^	0.05 ± 0.00 ^abcdeh^	0.11 ± 0.01 ^abcfgi^	0.05 ± 0.00^abcdeh^	0.03–0.11
Se	0.02 ± 0.00 ^bcdefghi^	0.01 ± 0.00 ^acdefghi^	0.03 ± 0.00 ^abegi^	0.03 ± 0.00 ^abegi^	0.04 ± 0.00 ^abcdfgh^	0.03 ± 0.00 ^abegi^	0.05 ± 0.00 ^abcdefhi^	0.03 ± 0.00 ^abegi^	0.04 ± 0.00 ^abcdfgh^	0.01–0.05
Mo	0.26 ± 0.01 ^bcdefghi^	0.39 ± 0.02 ^acdfghi^	0.34 ± 0.02 ^abdefghi^	0.60 ± 0.02 ^abcefghi^	0.42 ± 0.02 ^acdfhi^	0.53 ± 0.03 ^abcdeghi^	0.43 ± 0.02 ^abcdfhi^	0.77 ± 0.02 ^abcdefgi^	0.70 ± 0.03 ^abcdefgh^	0.26–0.77
Ag	0.10 ± 0.01 ^i^	0.10 ± 0.01 ^i^	0.10 ± 0.01 ^i^	0.10 ± 0.01 ^i^	0.10 ± 0.01 ^i^	0.10 ± 0.01 ^i^	0.10 ± 0.01 ^i^	0.10 ± 0.01 ^i^	0.45 ± 0.01 ^abcdefgh^	0.1–0.45
Sn	0.08 ± 0.01 ^cdeghi^	0.07 ± 0.00 ^cdefghi^	0.10 ± 0.00 ^abhi^	0.10 ± 0.01 ^abhi^	0.10 ± 0.01 ^abhi^	0.09 ± 0.01 ^bhi^	0.10 ± 0 ^abh^	0.13 ± 0.01 ^abcdefgi^	0.05 ± 0.00 ^abcdefgh^	0.05–0.14
Ba	1.21 ± 0.08 ^bcdefghi^	2.48 ± 0.07 ^acdefghi^	3.86 ± 0.27 ^abdefghi^	5.39 ± 0.27 ^abcefghi^	6.3 ± 0.44 ^abcdfghi^	1.97 ± 0.14 ^abcdei^	1.9 ± 0.11 ^abcdeh^	2.12 ± 0.13 ^abcdegi^	1.73 ± 0.1 ^abcdefh^	1.21–6.3
Tl	<0.01	<0.01	<0.01	<0.01	<0.01	<0.01	<0.01	<0.01	<0.01	<0.01
Pb	0.26 ± 0.01 ^abcdefghi^	0.39 ± 0.02 ^acdefghi^	0.63 ± 0.03 ^abdefhi^	0.76 ± 0.05 ^abcfghi^	0.81 ± 0.05 ^abcfghi^	1.05 ± 0.05 ^abcdeghi^	0.65 ± 0.05 ^abdefhi^	0.54 ± 0.03 ^abcdefgi^	0.96 ± 0.03 ^abcdefgh^	0.26–1.05

^1^ Interval of variation. The letter a in superscript indicates a value significantly different compared to the PL-1 variety, b compared to PL-2 variety, c compared to PL-3 variety, d compared to PL-4 variety, e compared to Tw variety, f compared to N2 variety, g compared to N3 variety, h compared to N4 variety, i compared to N5 variety.

**Table 8:** Nutritionally essential trace element content of 9 food-grade sorghum inbred varieties grown under typical Mediterranean conditions (μg kg^−1^).

Parameter	U	Sb	Hg	Cd
PL-1	10.01 ± 0.70 ^bcdefghi^	3.0 ± 0.2 ^bcdefhi^	46.5 ± 1.8	112.5 ± 3.3 ^bcdefghi^
PL-2	25.27 ± 1.52 ^acdefghi^	8.0 ± 0.5 ^acdefghi^	47.0 ± 3.2	25.0 ± 0.7 ^acdefghi^
PL-3	38.26 ± 1.15 ^abdefghi^	5.5 ± 0.3 ^abdefgi^	47.0 ± 2.8	27.0 ± 1.3 ^abdefghi^
PL-4	49.41 ± 2.47 ^abcfghi^	13.5 ± 0.5 ^abcefghi^	49.0 ± 3.4	15.5 ± 0.6 ^abcefghi^
Tw	54.11 ± 3.79 ^abcfghi^	6.0 ± 0.1 ^abcdfghi^	46.5 ± 2.3	13.0 ± 0.6 ^abcdfghi^
N2	15.09 ± 1.06 ^abcdegi^	2.5 ± 0.1 ^abcdeghi^	46.0 ± 2.3	17.0 ± 0.5 ^abcdeghi^
N3	33.6 ± 2.02 ^abcdefhi^	3.0 ± 0.1 ^bcdefhi^	47.1 ± 1.8	48.0 ± 2.8 ^abcdefhi^
N4	15.28 ± 0.76 ^abcdegi^	5.5 ± 0.2 ^abdefgi^	47.5 ± 1.43	42.0 ± 2.1 ^abcdefgi^
N5	8.83 ± 0.44 ^abcdefgh^	<0.1 ^abcdefgh^	47.7 ± 2.82	379.5 ± 26.5 ^abcdefgh^
Int.Var ^1^	10.01–54.11	<0.1–13.5	46.0–49.0	13.0–379.5

^1^ Interval of variation. The letter a in superscript indicates a value significantly different compared to the PL-1 variety, b compared to PL-2 variety, c compared to PL-3 variety, d compared to PL-4 variety, e compared to Tw variety, f compared to N2 variety, g compared to N3 variety, h compared to N4 variety, i compared to N5 variety.

**Table 9:** Mineral content of the sorghum grains was compared to Recommended Dietary Allowance/Adequate Intake (RDA/AI) for these minerals. Additionally, a comparison was made with the findings from Jaćimović et al. (2023) [30].

Mineral	mg/100 g Sorghum	US RDA/AI	Jaćimović et al., 2023 [30]
Mg	0.6–0.8	400 (adult males age < 50)	57.6–92.7
Fe	11.3–19.4	18 (adult males and females age > 50)	1.4–3.4
K	1.3–1.6	3400	96.4–232.3
Zn	9.4–57.8	11 (adult males)	1.3–2.5

**Table 10:** Gliadin content (as ppm) in flours by using sandwich R5 enzyme-linked immunosorbent assay (ELISA).

Sorghum Inbreds	Content (ppm) ^2^
PL-1	<5
PL-2	<5
PL-3	<5
PL-4	<5
Tw	<5
N2	<5
N3	<5
N4	<5
N5	<5
Wheat gliadin standard ^1^	56

^1^ Gliadin standard from wheat (Sigma). ^2^ Mean values from 3 measurements.

## Data Availability

The original contributions presented in the study are included in the article. Further inquiries can be directed to the corresponding author/s.
